# Compulsory treatment in patients’ homes in the Netherlands: what do mental health professionals think of this?

**DOI:** 10.1186/s12888-020-02501-7

**Published:** 2020-02-24

**Authors:** D. A. de Waardt, F. M. M. A. van der Heijden, J. Rugkåsa, C. L. Mulder

**Affiliations:** 1 Department of Psychiatry, ETZ Hospital (Elisabeth-TweeSteden Ziekenhuis), Hilvarenbeekseweg 60, Tilburg, 5022 GC the Netherlands; 2 Vincent van Gogh for Mental Health, Venray, the Netherlands; 3 Health Services Research Unit, Akershus University Hospital, Lørenskog, Norway; 4 Centre for Care Research, University of South-Eastern Norway, Porsgrunn, Norway; 5 Parnassia Psychiatric Institute, Rotterdam, the Netherlands; 6 Department of Psychiatry, Epidemiological and Social Psychiatric Research institute (ESPRi), Erasmus MC, Rotterdam, the Netherlands

**Keywords:** Compulsory community treatment, Outpatient commitment, Mental health law

## Abstract

**Background:**

Compulsory treatment in patients’ homes (CTH) will be introduced in the new Dutch mental health legislation. The aim of this study is to identify the opinions of mental health workers in the Netherlands on compulsory community treatment (CCT), and particularly on compulsory treatment in the patients’ home.

**Methods:**

This is a mixed methods study, comprising a semi-structured interview and a survey. Forty mental health workers took part in the semi-structured interview about CCT and 20 of them, working in outpatient services, also completed a questionnaire about CTH. Descriptive analyses were performed of indicated (dis) advantages and problems of CCT and of mean scores on the CTH questionnaire.

**Results:**

Overall, the mental health workers seemed to have positive opinions on CCT. With respect to CTH, all mean scores were in the middle of the range, possibly indicating that clinicians were uncertain regarding safety issues and potential practical problems accompanying the use of CTH.

**Conclusions:**

The majority of the participating mental health workers in this study had a positive attitude towards CCT, but they seemed relative uncertain about potential possibilities and problems of working with CTH.

## Background

Considerable controversy is raised around the world by compulsory treatment in the community for people with psychiatric disorders. The Dutch government is about to implement new legislation that allows compulsory treatment in the community (CCT) to be extended to patients’ homes (CTH), with the implication that patients can be physically forced to undergo treatment at home.

CCT allows people with psychiatric illnesses to live at home under certain conditions, on the understanding that they can be readmitted to hospital if these conditions are not met or if the patient’s condition deteriorates. The legal measure that allows for CCT is commonly called Community Treatment Order (CTO) or Outpatient Commitment. CCT was first introduced in the United States in the 1970s, and later in New-Zealand, Australia, Canada and many other, mainly Western, countries [1–3]. CCT is considered a less restrictive alternative to involuntary admission and was implemented to prevent the frequent readmissions that can result from non-compliance with treatment [4].

Under current mental health legislation in the Netherlands there is an option for using CCT. CCT can be installed after the patient has been discharged from an involuntary admission, called ‘conditional discharge’, or “de novo” when psychiatric patients cause danger to themselves or others and this can be averted by complying with certain conditions while living in the community, for example accepting home visits and taking medication. In the context of CCT, involuntary treatment in patients’ home, such as using physical force when administrating depot-injection in the patient’s living room, is not allowed. However, the new Dutch mental health law introduces new powers for health professionals to force treatment – such as medication, physical restraint or blood tests – in patients’ homes, called “Compulsory Treatment at Home (CTH)”. As far as we have been able to establish, no other jurisdiction permits this.

CTH obviously raises many ethical and practical dilemmas. Should people be forced to take medication or undergo examinations, even in their own homes? How can we ensure that people receive the best of care at home? And what can be done if they do not comply with a treatment plan? This new form of compulsory treatment seems to go against the worldwide trend of efforts to reduce coercive interventions and the United Nations Convention on the Rights of Persons with Disability (UNCRPD) [5–7]. Also, there is no evidence that proves CCT is effective in reducing the number or duration of hospital admissions [8].

There are some studies internationally on what professionals think of CCT, they find generally positive attitudes towards CCT [9–13]. We could not find any studies that identify mental health care workers’ opinions on CTH.

The aim of this study was to learn what the opinion of psychiatrists and nurses working in mental health care in the Netherlands, is on CCT and the introduction of CTH. We also wanted to assess their views as to potential difficulties they think might arrive when CTH is implemented.

## Methods

### Aim and objectives

The aim of the study was to assess what mental health workers think of CCT and the introduction of CTH, and to learn what difficulties they expect might arise after the introduction.

### Design

We operationalised our research agenda in two parts, a mixed methods study, comprising a semi-structured interview and a survey. The semi-structured interview was used to ensure that participants could name any advantages and disadvantages. The survey was chosen to enable the use of a Likert Rating Scale.

We have asked psychiatrists and nurses to participate since these are the two professions within our mental health care system which are involved the most in treating severely mentally ill patients in the community while they are receiving CCT.

#### Part I

To assess opinions on CCT, we invited mental health workers, twenty psychiatrists working on a closed ward, ten psychiatrists working in community teams and ten nurses working in community teams, to a semi-structured interview that included two open ended questions:

- whether they were positive, negative or neutral towards CCT
- what they saw as its advantages, disadvantages and practical problems

We categorised the answers into separate advantages, disadvantages and problems. DW first categorised the answers to the semi-structured interviews and afterwards DW, FH and NM went through the different categories and answers and made a final list. We then counted how many participants raised each advantage/disadvantage/problem. Data saturation was reached after 16 interviews.

#### Part II

To assess views about CTH, short questionnaires were distributed to the 10 psychiatrists and 10 nurses who participated in part one and who worked in outpatient teams and had considerable experience of treating patients in a home setting. We choose to focus on psychiatrists and nurses working in an outpatient setting, since they are the ones who need to work with CTH in the home setting. Respondents where were asked to rate, using a 1–5 Likert ratio scale (1 = never, 5 = always) the likelihood that the following 9 given problems would arise when administering CTH.

Do you foresee problems:

- with the administration of forced medication?
- when tracing patients?
- concerning the possibility that patients’ homes will no longer feel safe to them?
- regarding the burden on the patients’ social networks?
- concerning the amount of staff needed?
- when collaborating with other stakeholders?
- concerning the amount of paperwork involved?
- with respect to staff safety?
- concerning the availability of hospital facilities?

This approach was similar to that used by Manning et al. [12] and Romans et al. [13] to assess clinicians’ attitudes towards community treatment orders. These problems were selected using these studies and a focus group. This focus group was held at a meeting with psychiatrists. At the focus group these papers were discussed and participants (*n* = 10) were asked to come up with other possible problems.

### Recruitment and sampling

We have included professionals from 12 different mental health care institutions from different parts of the Netherlands, which represents about 1/3 of the number of institutions that delivers this kind of care.

To ensure that the opinions of one team or one organisation would not heavily influence the results, two conditions on participation were applied: that no more than two participants from the same profession who worked at one location for the same health organisation could take part, and not more than one person from the same profession within the same team.

### Analysis

Data from Part I was analysed using content analysis. Data for Part II was analysed using the Statistical Package for the Social Sciences (SPSS V.24). The answers to the different items of the Likert ratio scales were analysed by calculating the mean score (and SD) for each item. They were then ranked based on their mean scores.

The Medical Research Ethics Committee at Erasmus University of Rotterdam deemed the study to fall outside the remit of the Medical Research Involving Human Subjects Act, and no further ethical approval was therefore required. We did not collect any identifiable personal data. Informed consent was obtained in writing from all participants. Data collection took place between December 2015 and May 2017.

### Availability of data and materials

The datasets used and analyzed during the current study are available from the corresponding author on reasonable request.

## Results

### Views and opinions on CCT

Forty participants (23 male and 17 female) participated in part I of the study. Thirty were psychiatrists, 20 of whom worked on a closed ward from which patients were regularly discharged on CCT, and 10 of whom worked with patients living in the community on CCT. Ten were nurses working in community mental health teams.

Their age ranged between 30 and 64 years. The average time they had been practising their profession was 9 years (range: 1–30). Thirty-five worked with adults with severe psychiatric disorders, one worked with adolescents, and four with elderly patients.

Overall, 29 of the 40 participants had a positive attitude towards working with CCT, 2 were neutral, and 9 held negative views.

The most often named advantages of an extension of CCT and introduction of CTH were: CCT potentially could avoid admission to hospital, could enable treatment to start at home sooner, and would be less restrictive and less invasive for patients than admission to hospital.

**Table:** 

*Some quotes of participants on the introduction of the extension of CCT in the Netherlands:* *“This new law (CCT and CTH) could be a way to prevent compulsory admissions, these can be very traumatising.”* “*I think the changes in legislation are a good thing, this way you don’t have to wait until things go wrong at home and then admit someone. You can actually start treatig someone at home before the damage is done.”* *“I think CCT is a good thing, if it is used to prevent harm, not to avoid admission to hospital.”* *“In theory it (CCT) is a nice idea, but I have my doubts about the practical exectution of CTH”.* *“The most important thing is to invest in a good therapeutical relationship, that way you may be able to avoid any compulsory treatment”.*	

The disadvantages most stated: CCT would limited patients’ autonomy, would put a strain on the therapeutic relationship, and it would put a burden on the patients’ social networks.

There were two themes that were often stressed by the participants:

- it is of major importance to invest in a good therapeutic relationship, since they felt that would give more change of a successful treatment than a court order would
- they felt the need for the government to invest in ways to improve psychosocial factors (like jobs and housing) for patients, since this would facilitate recovery probably more than new legislation would

### Problems anticipated with the use of CTH

**Fig. 1:**
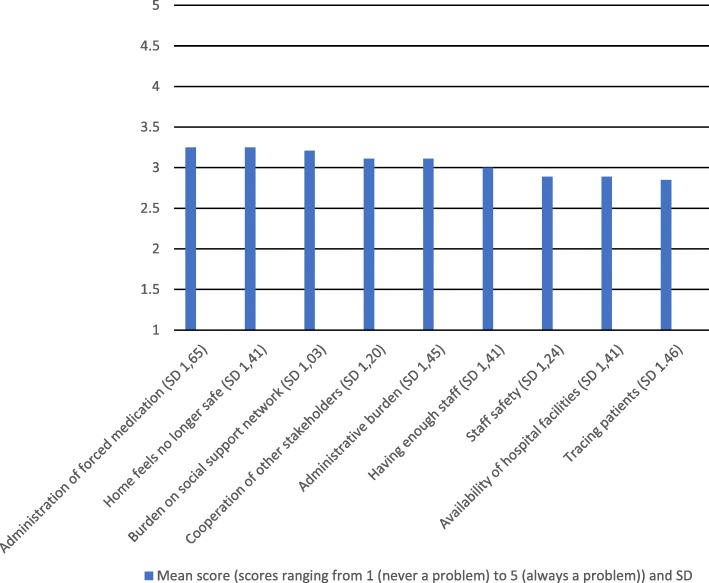
Healthcare workers’ opinions about the likelyhood of each of the following nine issues to occur during compulsory treatment at home (mean ratings)

Twenty participants, 10 psychiatrists and 10 community nurses, all working in outpatient teams took part. The items rated highest regarding potential problem areas of CTH were: the administration of compulsory medication at home and the possibility that patients would no longer feel safe in their own homes. The differences in scores between the potential difficulties were relatively small as shown in Fig. 1, and mean scores of all items were close to the mid-point of the scale.

## Discussion

When asked about their opinion, 29 out of 40 participants in our sample reported positive attitudes towards CCT. With respect to CTH, the administration of forced medication and the use of compulsion at home were rated as most likely to become problematic. However, as the mean scores of all items were close to the mid-point, we interpret this as a high degree of uncertainty in our sample as to how CTH will work in practice.

Other studies of mental health workers’ overall opinions of CCT have also identified generally positive attitudes. Coyle and colleagues [9] surveyed 288 community mental health workers working with patients receiving CCT. Eighty-three percent of psychiatrists and 67% of other professionals were in favour of working in a system with CCT as opposed to one without. Romans et al. [13] found that 78.8% of 202 psychiatrists in New Zealand and 84.8% of the 82 other participating mental health professionals preferred to work in a system with CCT. Manning et al. found that 60% of their 566 respondents preferred to work in a system using CCT [12], as did 62% of the 50 psychiatrists included in a Canadian study [10].

The positive attitudes towards CCT in the studies mentioned above and in our study stand in contrast to the evidence base: three large RCTs [3, 14, 15] and a Cochrane review [8] showed no effects, including no effects on compliance with treatment or reduction of the number of hospital admissions. A large meta-analysis by Barnett et al. [16] also showed no such effects, but suggested that CCT might have a positive impact on the use of community services and treatment adherence, though it is unclear whether this is because more services were offered. This finding was based on a small number of studies (15 and 5 respectively) none of which were RCTs.

The disadvantages of CCT identified by health professionals in these studies, such as use of compulsion, seemed to be considered to be outweighed by the advantages, such as facilitation of contact, medication compliance and early identification of relapse [4, 11–13]. It should be borne in mind that the circumstances of all these studies were different from those that will apply under the new Dutch legislation. In these studies, “compulsion” usually means an obligation on patients to take medication that may be enforced by a readmission, but not by physical force in the home of patients. Nevertheless, research from a number of countries shows that patients on CCT do feel coerced into taking their medication at home [17].

Our study also investigated the view of mental health professionals on compulsory treatment in patients’ homes. Our data identified a contrast between positive views about CCT in general (as reported elsewhere) and uncertainty about CTH. For example the use of physical force brings about new ethical and practical dilemma’s.

Given the scores on our questionnaire being close to the mid-point, it seems that the opinions of the psychiatrists and nurses were mixed and the participants were uncertain what to think about this new measure. They did not seem totally against this new measure, but also did not seem to perceive it as very promising. Once CTH is implemented in the Netherlands in 2020, further research into the experiences with CTH is necessary.

## Limitations

As this study had a relatively small number of participants, their views may not represent all mental health workers in the Netherlands. This amount does not allow for statistical analyses.

Also in this study only mental health care workers have been included and not yet service users or significant others. Because of this, only the opinion of one of the stakeholders has been evaluated and this might give a one sided perspective. The opinions of patients and their families will be the focus of a future study.

## Conclusion

Our study showed that while mental health workers in the Netherlands in general were in favour of CCT, they seem to have mixed or uncertain views regarding CTH.

In our view, it is paramount that when CTH is permitted as an extended form of CCT in the Netherlands as the first country in the world, a rigorous study should be conducted to establish exactly what the practical and ethical problems are, and who might benefit from this kind of legislation.

## Data Availability

The datasets used and analysed during the current study are available from the corresponding author on reasonable request.

## Abbreviations

CCT: Compulsory Community Treatment
CTH: Compulsory treatment in patients’ homes
CTO: Community Treatment Order
SD: Standard deviation
